# The effects of health shocks on family status: do financial incentives encourage marriage?

**DOI:** 10.1007/s10198-021-01319-8

**Published:** 2021-06-02

**Authors:** Andree Ehlert

**Affiliations:** Harz University of Applied Sciences, Friedrichstr. 57-59, 38855 Wernigerode, Germany

**Keywords:** Health shock, Marriage, Survivor’s pension, Widow, Old-age poverty, Unobserved heterogeneity, Frailty, Hazard model, SOEP, Germany, C40, D10, H55, I10

## Abstract

This paper asks whether marriage decisions of unmarried mature couples are driven by the prospect of financial advantages for the later widowed after one partner has suffered a serious health shock. We hypothesize that, in contrast to traditional marriage models, such health shocks may induce unmarried couples to obtain economic benefits, such as survivors’ pensions in particular, through marriage in advance of one partner’s death. This question has not yet been studied empirically. Hazard models capturing unobserved effects are applied to longitudinal data of the German Socioeconomic Panel. It turns out that the probability of marriage after male partners’ health shocks can increase significantly depending on the amount of expected survivors’ pensions for the (likely) surviving female partners. In contrast, an increased probability of marriage after health shocks to women (depending on the expected financial benefits to men) was not found. These findings are supported by various robustness checks. Economic and political implications are discussed and the results are placed in an international context.

## Introduction

In most Western countries and Western society, married couples (including registered partnerships) enjoy financial privileges such as splitting tariffs, inheritance tax exemptions, health care benefits, and (prospective) survivors’ pensions in contrast to the unmarried [21]. Consequently, marital status can have a strong influence on social security. This is particularly evident with respect to old-age poverty. It is well known that poverty rates for elderly people living alone are substantially higher than for couple households [17]. Especially within the group of elderly single people, previous marital status is an important factor associated with old-age poverty. For example, the poverty rate of unmarried (40–85 years old) German women in 2008 was 18% compared to a poverty rate of 14% for widows [15].^1^ The situation is accentuated by the social trend towards unmarried cohabitation [34].

Against this background, the paper asks whether unmarried (mature) couples in established relationships anticipate the economic benefits of marriage after health shocks which may indicate a decline in residual life expectancy. Couples therefore have an incentive to marry soon after the health shock, since payment of a later survivor’s pension is linked to prior formal marriage. From the economic perspective of adverse selection, this corresponds to the question of whether couples under asymmetric information tend to collectivize the risk of survivorship, since the health shock cannot be reliably questioned as a trigger for marriage after fulfilling a waiting period.^2^

To identify the economic motive behind the observable outcome (i.e., marriage), we make use of the amount of the prospective survivor’s pension (as well as saved inheritance tax for married couples for real estate and assets) that is lost in case of non-marriage. These numbers can be estimated from the data. Besides a possible moral component, the decision for or against marriage after health shocks primarily reflects individual economic considerations. These involve weighing up the benefits of virtually free survivors’ insurance (and inheritance tax savings) through marriage against the associated uncertain financial obligations associated with legal marriage (e.g., risk of the partner surviving longer than expected with mutual maintenance obligations such as long-term care). We study whether people are aware of this bargain and how marriage after health shocks depends on the prospective level of future financial benefits.

Of course, the identification of this assumed link between health shocks and marriage for financial reasons depends on numerous assumptions and limitations, which are carefully discussed in the paper. For example, legal requirements to prevent such pension marriages (*marriages for maintenance*) can completely exclude the payment of survivors’ benefits. In addition, the amount of the assumed pensions and inheritances, the calculation of which is very technical, is difficult to forecast in advance from an individual perspective. Another concern is the appropriate severity of a health shock to trigger a *pension marriage* (whereby too light health shocks are not associated with reduced life expectancy and too severe shocks may completely suppress all thoughts of marriage).

So far, the economics of marriage literature has concentrated on traditional partnership role models, which focus on matching partners at a young age to increase the productivity of the joint household (e.g., through risk-sharing, substitution of household inputs, spillover effects of social status, etc.), see e.g., [7]. This process is largely ignored in our analysis. Instead, the focus here is on a later stage in the partnership life cycle with an established, unmarried relationship.^3^ In addition, we limit our sample to mature couples (at least one partner of age 45 or over) to ensure sufficient sensitivity to pension issues. Our empirical analysis is based on hazard models with time-varying covariates using data from the German Socioeconomic Panel (SOEP). This allows us to account for (time invariant) unobserved heterogeneity and, hence, to offset potential bias caused by unmeasured variables such as physical attractiveness or previous marriage experiences.

This paper integrates with a large existing (health economics) literature on the interplay between partnership and health status. It extends this literature by empirically analyzing financial incentives to marry after health shocks for mature couples. The existing literature can be classified first by whether health is considered an influence variable (selection theory, i.e., changes in health precede changes in relationship status) or outcome (causation theory, i.e., the relationship status itself affects health) of a partnership, see, e.g., [30] for an extensive discussion of the underlying literature. Our paper can be placed in the first domain, considering a sample of stable (cohabiting), mature, and unmarried couples. The majority of the existing literature tends to focus on how a health shock has a (de)stabilizing effect on partnerships. For example, [10] examine the impact of gender-specific health shocks on the persistence of partnerships with data from the SOEP. They account for possible reverse causality and show that mental health shocks have a significant negative impact and physical health shocks (in some model specifications) can have a positive impact on partnership (and marriage) stability.^4^ One of the few studies that, similar to our analysis, investigate the transition from cohabitation to marriage as a function of (here mental) health is [42]. Using data from the British Household Panel Survey, they find a significant effect of a 12-item self-reported mental health on cohabitation dissolution, but mixed effects on marriage out of cohabitation. It is precisely this latter point to which our study connects, as we examine the influence of health shocks on the transition from cohabitation to marriage (and thus a stabilized partnership). Nevertheless, it should be emphasized that our research question is different. It is less about the continuation or intensification of the relationship in relation to the health shock, but about financial incentives after a health shock in the form of survivors’ pensions (and tax benefits), which has so far been excluded from the empirical literature. To separate this financial motive from moral (and other) reasons for marriage, identifying these financial incentives [discussed in “Marriage and Health” as *income at risk* (IAR) and *assets at risk* (AAR)] in conjunction with the health shock is a main focus of this paper.

A further connection of our study to the existing literature can be seen in [28], who, similar to our study, consider the transition of couples to marriage (and further transitions in relationship status) as a function of health status with a hazard model for longitudinal data from the Netherlands. No significant association is found for subjective health with marriage, but conversely, a significant positive effect of subjective health problems on divorce of existing marriages. Despite the methodological similarities to our study, financial incentives in the transition between relationship statuses (as well as unmarried couples over 65, which play a major role for our question) are not taken into account here either. Finally, our analysis also complements the literature on marriage as a risk pooling instrument, see, e.g., [47]. This is because marriage further increases the existing risk protection of a stable but informal partnership when the couple’s risk situation changes due to the health shock of a partner. From this perspective, our question is whether people access the (largely free) insurance contract *marriage* more quickly when its payout (in the form of a survivor’s pension and tax benefits) becomes more likely. The commonly observed selection of healthy partners into marriage, cf. [28], is thus reversed into an adverse selection. Unlike, for example, [33], who justify adverse selection into marriage with its protective effect for health, however, our results show that it is not the health shock per se that drives marriage. Rather, it is the increased risk with the health shock of losing financial benefits of marriage due to the premature death of the partner. Further examples for the analysis of people’s marriage propensity in response to economic incentives include [3, 4] who report that for aggregate U.S. data tax incentives may increase the likelihood of marriage (albeit with a low elasticity of approx. − 0.05, i.e., taxes would have to fall by 20% for a 1% increase in the marriage rate). By contrast, [13] finds that taxes (and welfare benefits for the unmarried) have a significant effect on the probability of separation.

The remainder of this paper is organized as follows. The section “Marriage and health” briefly discusses the economics of marriage. Details of the German survivor’s pension system are covered in “Social security for widow(er)s”. The section “Data and methods” presents the database and econometric methods. This is followed by the section “Results and discussion”. Conclusion is drawn in the final section.

## Marriage and health

Economic principles such as utility maximization, household production functions, or the notion of marriage market equilibria have entered the modeling of marriage ever since the seminal work of Becker [7, 8]. The empirical analysis applied in this paper, instead, focuses on the transition from cohabitation (as an indicator for a stable partnership) to legal marriage.

However, the effects of Becker’s traditional marriage-related factors (such as labor force participation, income and education, desire to have children) can be reduced, since the matching phase of the partners can be regarded as (largely) completed by living together.^5^ We still assume that the influencing factors of classical marriage models play a role—albeit a smaller one—in our model (since an upcoming wedding event will also depend on very traditional considerations, even for long-standing, mature couples). We therefore start with Becker’s classical approach and make the following adjustments.

From an economic perspective, a long period of unmarried cohabitation means that a couple’s marriage incentives had been too low to trigger their legal marriage, so far. A health shock, however, may change the picture when associated with a prospective income stream and, hence, may compensate for lack of other marriage-promoting factors. More precisely, to disentangle the social security motive for legal marriages of mature couples, we introduce the concept of a partner’s IAR. The IAR reflects the social security component (survivor’s pension) which the surviving unmarried partner loses when the other partner dies before marriage. Note that the IAR alone, however, is not sufficient to fully reflect people’s economic windfall considerations with respect to a surviving partner’s social security. It rather must be seen in the light of its relevance, i.e., the probability of the other partner’s death (reduced residual life expectancy) after his or her health shock.

We approximate the latter by a *severe* health shock (to be defined in the section “Main variables”). We hypothesize that a deadweight effect exists if a higher IAR after severe health shocks increases the marriage hazard for cohabiting mature couples. Put differently, we hypothesize that unmarried cohabitation would have continued if there had been no health shock. In economic terms, we ask whether the disadvantages of a person’s bad risk aspects (i.e., his or her poor health) are over-compensated by the advantages of the good risk aspects associated with the prospect of a future survivor’s pension. As already noted in the section “Introduction”, our approach may indeed imply a reversed impact of the factor health on the marriage hazard as compared to Becker’s model in which poor health indicates a reduced household production capacity. In our context, however, health shocks serve as a trigger event which indicates the possibility to take advantage of financial benefits (survivors’ pensions) in the future.

Still, even for mature couples, a pure *marriage for maintenance* strategy is far from dominant and, hence, poses an interesting economic decision. The reason is that legal marriage involves far-reaching maintenance obligations affecting personal wealth. This may attenuate the benefits of prospective survivor pensions and tax privileges in view of high costs for a moribund partner’s long-term care, changes in legal succession, unemployment benefits being offset against spousal income, and also loss of former survivors’ pensions upon remarriage. For example, spouses are generally obliged to care for their partner’s living expenses and to (partially) bear nursing home costs (cf. e.g., §§1360, 1360a German Civil code, BGB) with monthly co-payments in 2019 amounting to EUR 1891 on average [54]. So far, it has not been analyzed empirically before if, in the eyes of the people, the potential economic benefits outweigh the above risks.

## Social security for widow(er)s

German law provides mutual maintenance obligations through work or capital for spouses and registered life partners (see §1360 BGB and § 5 LPartG, German Civil Partnership Act, which was replaced by the introduction of same-sex marriages in late 2017). To compensate for the loss of maintenance after the death of a partner, the surviving spouse is entitled to a survivor’s pension. Details of the rules are provided in § 46 SGB VI (Social Security Code, book six) for statutory pension schemes and in the Civil Service Pensions Act (Beamtenversorgungsgesetz), respectively.^6^ A brief discussion of the relevant regulations in other countries follows in the section “Discussion”.

As a general prerequisite to qualify for a survivor’s pension (either statutory or for civil servants), waiting periods of up to 1 year after marriage were introduced. By doing so, legislators anticipated people’s private information, i.e., their assumed attempt to take advantage of the financial benefits of marriage in the short term.^7^ However, the rule does not apply if the surviving partner is able to disprove that the marriage was purely motivated by financial considerations. Furthermore, the survival time after the initial diagnosis of common, severe widespread diseases such as cancer or heart failure has increased far beyond the usual 1-year waiting period, so that legal sanctions would not be effective. For example, 5-year relative survival in Germany amounts to 62.2% (61.7–62.8%) for colon cancer, to 83.6% (83.2–84.0%) for breast cancer, and to 89.4% (88.8–89.9%) for prostate cancer (European mean age-standardized, 95% confidence intervals in brackets, see [11]). Even for lung cancer, recent data report a 1-year relative survival of more than 40%, cf. [45]. One may argue that such prolonged survival times upon diagnosis of a life-threatening illness may gradually weaken the above 1-year waiting-time and, hence, make the question of marriages for maintenance seem even more relevant.

Further prerequisites to qualify for a survivor’s pension include a minimum social security contribution period of 5 years for the deceased spouse (with exceptions, e.g., in case of work accidents). Also, the surviving spouse may not have remarried, although a pre-existing survivor’s pension may be reinstated in case of divorce.^8^ Under these conditions, a so-called lower rate statutory survivor’s pension at 25% of the deceased partner’s (calculated) pension is paid as a subsidy for maintenance for a 2-year period (whereas it is paid indefinitely for cases prior to 2002).

By contrast, the higher rate statutory survivor’s pension is intended as a (full) compensation of the deceased partner’s maintenance. It amounts to 55% of the deceased partner’s (calculated) pension (60% for marriages concluded prior to 2002 with at least one partner born before 1962) and is paid indefinitely (except after remarriage). Prerequisites for the higher rate pension are more stringent to ensure that surviving spouses cannot be expected to provide for their own maintenance themselves. To qualify, either a minimum age of 45 years and 7 months (as of 2018, gradually increasing to 47 years until 2029), or a reduced earning capacity, or raising a child is required in addition to the above general conditions.

Note that own income is partly offset against survivors’ pensions. Roughly speaking, payments will be reduced by a 40% share of own (net) income exceeding a personal income allowance (of approx. EUR 800 in 2018, supplemented by child allowances).

Provisions for civil servants are similar except for different (generally more generous) regulations for offsetting own income against the survivor’s pension depending on the deceased person’s salary grade. Also, some rather technical rules apply for deceased spouses who are 20 or more years older than the surviving partner. In this case, the pension will be reduced by a certain percentage which, however, may be offset for long-term marriages, see § 20 BeamtVG for details.

Empirically, some 86% of widows aged 65 and over are entitled to a statutory survivor’s pension of approx. EUR 675 per month (EUR 300 for widowers), see [32]. Note that survivor’s pensions are higher than own pensions for approx. 19% of all widows and, hence, amount to their main income source, whereas the same share for widowers is only 0.1% (based on SOEP data). All in all, the statistics in [12] show that about 4.9 million widows and 0.6 million widowers received a statutory survivor’s pension in 2010. Most of them were of the higher rate type (98.9% for women and 97.8% for men). Own income was offset against 29% of survivor’s pensions (78% for widowers).

## Data and methods

### The data

This study is based on longitudinal data obtained from the SOEP for the time period 1994–2017, cf. [48]. The SOEP is an annual survey panel started in 1984. For the year 2017, it comprises approx. 32,500 individuals living in about 19,800 households in Germany.^9^ It is based on separate interviews with each household member aged 17 and over, i.e., all data used in this study are self-reported. Core questions of the SOEP comprise, for example, demographic aspects, marital status, qualification, occupational status, income, housing, and health.

In this study, spells for couples are identified based on person-level SOEP data (which include respective partner IDs, if applicable). Our sample is derived from the raw SOEP data in four main steps. The data will (1) be limited to observations from 1994 onward, as data on self-reported health have only been available since then. As our main interest lies in the legal act of marriage as opposed to pure partner matching, we (2) restrict our sample to unmarried stable partnerships of mature couples (i.e., at least one partner of age 45 or over). As an approximation, an unmarried stable partnership is defined by the time between the couple’s moving in together (as indicated by the SOEP) and the legal marriage (if applicable), cf. “Marriage and health”. To avoid left censoring the sample is (3) restricted to those couples whose dates of moving in together are observable.^10^ Otherwise, the duration of the stable partnership (which corresponds to being at risk of marriage and which clearly influences marriage hazards over time) would be unknown. These restrictions (the robustness of which will be tested in Appendix 7) result in a preliminary sample of 880 couples and 3021 couple-years.^11^ Finally (4), all pair-years with missing values for the covariates discussed in the section “Main Variables” are excluded, resulting in a sample with 795 couples and 2767 couple-years. Table 1 summarizes this sample in terms of a *life table* for unmarried couples. For each year from the start of an unmarried partnership (cohabitation), the table shows how many couples married during the corresponding year as well as the total number of couples. For row 1, this means that, of course, all 795 couples in the sample cohabited in the first year and 229 of them married during the year. These 229 couples are not counted in row 2, because their *marriage* endpoint was reached and they leave the sample. For 2 years (row 2), only a total of 487 couples from the sample then lived together unmarried, of which 34 married during the year. (This is 79 fewer couples than after subtracting the married ones from row 1, since some couples left the SOEP for other reasons). The other rows should be interpreted accordingly. In total, 2767 couple-years are observed over the sample period of a maximum of 24 years.^12^ Note that the mean duration of the cohabiting partnership before marriage amounts to 2.70 years (with a s.e. of 0.17) which is roughly in line with prior empirical findings from the literature, see e.g.,  [22, 23]. By contrast, the mean duration of cohabiting partnerships until separation or sample attrition amounts to 5.75 years (s.e. of 0.10).

**Table 1:** Partnership life table

Year of relationship (cohabitation)	Marriage	Not married	Total
1	229	566	795
2	34	453	487
3	31	365	396
4	16	301	317
5	14	235	249
6–24	472	51	523
Total (all partner years)		375	2767

Cohabiting couples from the time of moving in together with at least one partner of age 45 years or over. Marriage events (column 2) are broken down per duration of the partnership

### Main variables

Table 2 gives an overview of descriptive sample characteristics (by couple, with separate values for male and female partners in the upper part) for key socioeconomic variables related to people’s marriage decisions, cf. “Marriage and health”. In a first step (not shown), the mean values over time were calculated for all 795 couples in the sample (depending on the duration of their stay in the sample), cf. “The data”. The table then contains as a second step the means and standard deviations over all these $N=795$ values. A discussion for selected variables to be included in our empirical model is given below (with a special emphasis on the potential drivers of marriage decisions of mature couples).

**Table 2:** Basic sample characteristics

Variable	Male		Female		$N$
	Mean	Std. Dev.	Mean	Std. Dev.	
Age	52.95	10.08	49.16	10.28	795
Recently unemployed ($\ge$ 4 months)	0.10	0.26	0.11	0.28	795
Total unempl. exp. (months)	1.36	3.29	1.48	3.29	795
Education (years)	12.19	2.67	11.94	2.53	795
Child born prev. year	0.02	0.14	0.02	0.11	795
Non-German	0.08	0.27	0.10	0.29	795
IAR (1000 EUR, 2011)	2.49	2.83	5.79	5.15	795
Civil servant (yes/no)	0.06	0.23	0.03	0.18	795
Widowed before (yes/no)	0.08	0.26	0.12	0.33	795
Divorced (yes/no)	0.28	0.45	0.31	0.46	795
Current health status (1 = very good, 5 = bad)	2.70	0.87	2.79	0.87	795
Health shock	0.06	0.18	0.07	0.21	795
Couple-level					
Distance city center (km)	21.99	22.88			795
Property owners (yes/no)	0.41	0.48			795
No. of children	0.47	0.82			795
Couple’s income (1000 EUR, 2011)	52.09	35.54			795
Income difference m-f (1000 EUR, 2011)	11.40	24.53			795
Main earner female (yes/no)	0.28	0.41			795
Marriage (uncensored obs.; yes/no)	0.29	0.46			795

- **Labor market participation:** Two variables, namely the current (this or last year) unemployment of at least 4 months and the total experience of unemployment (in months), are included in our empirical model. Note that labor market participation for women is proposed to have a decreasing effect on marriage rates which may be based on a shadow price argument for household production in Becker’s model. Empirical evidence for this proposition is mixed, however, see, e.g.,  [40]. In general, the empirical impact of unemployment on marriage is largely ambiguous a priori, see [2, 3].
- **Education:** This variable (schooling and education in years) is included to reflect the labor market opportunities and economic attractiveness, but also the socioeconomic status of a person. It can be reasonably assumed that this has an influence on the probability of marriage, but the sign is still unclear from an empirical point of view. For example, for U.S. data college education is found to increase marriage probability, see e.g.,  [41], whereas women with a college degree have a significantly reduced marriage probability as discussed in [47]. Moreover, in a sample of Germans, years of education significantly reduce the probability of marriage (at age 35), see [35].
- **IAR:** Recall from “Marriage and health” that the empirical strategy of this study is largely based on the so-called IAR which reflects the opportunity cost of non-marriage with respect to survivors’ benefits. The IAR thus captures the difference between the expected survivor’s pension in the case of marriage and that of non-marriage from the perspective of the surviving partner. Note that the male partner’s IAR includes the female partner’s pensionable income components and vice versa. For example, Table 2 reflects that, empirically, male pensionable income (i.e., the female’s IAR) is more than twice as much as female pensionable income. The legal provisions discussed in the section “Social Security for Widow(er)s” are considered to quantify the annual amount of the IAR. For example, accrued pension claims are adjusted for age and previous survivors’ pensions. Furthermore, own above-threshold income is offset against prospective survivors’ pensions. Some simplifications are necessary, however, as detailed calculations depend strongly on the circumstances of the individual case (e.g., transitional regulations, qualifying periods or child allowances). The simplifying assumptions are however critically reviewed in Appendix 7.
- **Health:** The definition of health shocks is based on yearly self-assessed five point Likert scale ratings as no clinical data are included in the SOEP (current health status, 1 = very good, 5 = bad). A two-point decrease in the previous year or the year before will be considered a health shock in our empirical model. Using this definition, approximately 4% of men and 5% of women (within all couple-year observations in the sample), respectively, experience a health shock. These numbers are fairly in line with the sample proportions of health shocks (although using different health indicators) discussed in [10]. In addition, our definition of a health shock is guided by the rule proposed in [37] to use a drop of two standard deviations of subjective health (corresponding to a two-point decrease in our data), as a health shock should be serious enough to make people at least think about their partner’s financial provision after their own death.
  Since all these measures are subjective health indicators, the relevance of the relationship between subjectively perceived and objective health for the results of our analysis should be briefly discussed. Note, however, that for our problem, the aim is not to record a health shock as objectively as possible in the sense of a clinical diagnosis. An ideal health shock indicator for our research question should be able to capture subjective residual life expectancy. Indeed, it is precisely this subjective life expectancy that can be considered as a predictor of important life decisions (which include caring for one’s partner after one’s own death), cf. [18]. There, it is also shown that self-reported health is significantly positively associated with subjective life expectancy.^13^ Moreover, the use of a subjective health indicator for our research question can be justified by the results in [31]. There it is shown that subjective health shocks are positively associated with, on the one hand, having a limited subjective residual life expectancy (*limited future time perspective*) and, on the other hand, with increasing generativity motives (which include survivor care). If anything, existing empirical studies suggest a possible overestimation of subjective residual life expectancy after health shocks, which could limit the significance of our results in the sense of a downward bias, cf. [39, 43].
  Our strategy to address the problem involves two points in particular. First, we will at least partially mitigate the potential bias using an alternative indicator of satisfaction with health, since satisfaction more than current health requires personal assessment also against the background of medical circumstances, cf. the robustness checks in Appendix 7. Here, relatively high thresholds of decline in health satisfaction are used, which excludes smaller fluctuations in satisfaction with health and increases the probability of detecting serious health shocks. Second, Appendix 7 also resorts to a more objective health indicator in the form of the number of hospital nights to define health shocks.
- **Income:** Various dimensions of income such as the level of pre-marriage individual income, income differentials, income sources, individual and joint income after marriage, as well as taxation have been discussed extensively in the marriage literature. For example, a positive effect of income on marriage (but only a small effect for transitions from cohabiting unions to marriage) is described in [41] based on U.S. data from 1979 to 1993. Results of [1] and [49] point towards the same direction. In our empirical model, the couple’s income includes labor, self employed, and capital income. As the latter is only available at the household level, it is disregarded for the male-female income difference. All income variables are inflation-adjusted in terms of 2011 prices [51].

### Econometric model

We model a discrete time hazard function for the transition of unmarried couples to the state of marriage (conditional on time-varying covariates). The discrete-time representation reflects the availability of yearly interval data for most of the covariates in the underlying SOEP dataset. Estimated model parameters will then be used to test our hypothesis that health shocks increase the marriage propensity subject to prospective survivors’ pensions, cf. “Introduction”.

A vast literature has applied survival models to questions related to marriage and fertility before. For example, [38] apply such models to fertility data from Costa Rica. Among the first studies to use proportional hazard models for marriage timing is [53]. In [49], a semiparametric Cox proportional hazards model is proposed to study the effect of risk attitude on time to marriage. Similarly, [47] uses a logistic hazard model for the effect of people’s risk tolerance on timing of marriage. The question of how labor market conditions affect the timing of marriage in Spain is analyzed via a semiparametric Cox approach in [19]. Different survival models for the marriage process using German and U.S. data are discussed in [14]. In the context of health shocks and people’s subsequent reactions, discrete-time hazard models are applied, e.g., to smoking behavior and diet [52], and retirement [20].

In this study, the observation units of interest are couples $i= 1,\ldots ,N$ who change to the state of marriage at year $j=0,1,2,\ldots$ after having entered into a committed relationship (which we conceptualize by moving in together). We focus on couples being at risk of marriage at the beginning of the sample, i.e., we apply stock sampling as in [24]. According to the available data, the analysis is based on disjoint yearly time intervals

$$\begin{aligned}{}[a_1, a_2), [a_2,a_3),\ldots ,[a_{M-1},a_M). \end{aligned}$$

Here, the date $a_j$, $j=1,\ldots ,M$, represents the first day of the *j*th calendar year following a couple’s sample entry (i.e., the year of moving in together for an unmarried couple). The last interval represents marriage or censoring at time *M* whereafter the couple leaves the sample (note that we suppress the couple-index *i* for convenience). To avoid left censoring, we restrict the sample to those partnerships whose beginning (moving in together) is observed, i.e., ongoing partnerships with unknown start date are dismissed. A couple’s potential marriage is modeled as a permanent (absorbing) state, such that our data are of the single spell type. Consequently, couples who do not marry before exiting the sample are considered as (right) censored.

The hazard rate is assumed to take the complementary log–log form, i.e., a discrete-time analogue of the standard continuous time proportional hazard model [24, 26, 36]

1 $$\begin{aligned} h_j(X_{ij}) = 1 - \exp (-\exp [\beta ' X_{ij} + \gamma _j + \log (\varepsilon _i)]), \end{aligned}$$

where $X_{ij}$ are (vectors of) time-varying covariates for couple *i* at time *j* and $\gamma _j$ represents the baseline hazard. The vector $\beta$ includes the corresponding coefficients to be estimated. Couple specific (time invariant) unobserved heterogeneity (*frailty*, i.e., a couple’s unobserved propensity to marriage not covered by *X* variables) is modeled parametrically by a random variable $u_i$ to be specified in the section “Main results”. This frailty term includes, e.g., attitudes, cultural imprints, or personal experience that will likely affect time to marriage. If such unobserved variables are ignored in the hazard model but are indeed correlated with some of the covariates, parameter estimates are likely to be biased. For example, it has been found that negative duration dependence will be overestimated, while coefficients of covariates will be underestimated (in absolute terms). See [26] for a more detailed discussion, and [27] for the potential consequences of unobserved heterogeneity in a health economic context.

In addition to the complementary log–log model in Equation (1), we propose a binary dependent regression model for comparison as its coefficients have a somewhat simpler interpretation and turned out comparable to the results of the hazard model in many applications. The standard latent model reads

2 $$\begin{aligned} Y_{ij}^*=\beta ' X_{ij} + \alpha _i + u_{ij}, \end{aligned}$$

where $Y_{ij} = 1$ if $Y^*_{ij}>0$ and $Y_{ij} = 0$, else, with a symmetric distribution for $u_{ij}$ (leading to logit or probit, for example), cf. [55] for details. Here, $Y_{ij}$ indicates whether couple *i* is married in period *j*. Unobserved individual heterogeneity is captured by $\alpha _i$ treated as random or fixed effect. Note, however, that in contrast to the above hazard approach, the binary dependent regression models whether couple *i* makes a transition to marriage in year *j* at all (during the observation period) or not. In other words, the information contained in the passage of survival time is completely discarded in this model.

## Results and discussion

### Main results

Results for the two models discussed in the section “Econometric model” are presented in Table 3. Coefficient estimates for male partners are given in the upper part of the table, followed by the results for female partners in the middle part, and estimates for couple-level coefficients in the lower part. The hazard model given in Eq (1) was implemented using the pgmhaz8 routine in Stata/MP 13.1 (left-hand side of the table), cf. [25, 50]. Here, a monotonic baseline hazard is modeled by a parametric Weibull specification.^14^ The frailty term is modeled by a gamma distributed random variable $\varepsilon _i$ with unit mean [where $u_i = \log (\varepsilon _i)$], cf. [25, 36].^15^ Results for the binary dependent regression in Eq. (2) were obtained by Stata’s built-in xtlogit routine using robust standard errors (right-hand side of the table).

**Table 3:** Coefficient estimates

Variable	Hazard (pgmhaz)		Logistic (xtlogit)	
	Coeff.	(Std. Err.)	Coeff.	(Std. Err.)
Male partner				
Age	0.07631	(0.06507)	0.14037	(0.09329)
Age2	− 0.00060	(0.00063)	− 0.00099	(0.00088)
Recently unemployed ($\ge$ 4 months)	− 0.13951	(0.25025)	− 0.14709	(0.32868)
Education (years)	0.00681	(0.02803)	0.02853	(0.03660)
Total unempl. exp. (months)	0.01876	(0.03220)	0.02807	(0.04602)
Child born prev. year	0.42278	(0.76073)	0.77842	(1.07485)
Non-German	0.74500*	(0.29583)	1.02671*	(0.44627)
IAR (m, 1000 EUR)	0.06797^†^	(0.03795)	0.11455*	(0.05044)
Civil servant (yes/no)	0.81014**	(0.29984)	0.94219*	(0.42808)
Widowed before (yes/no)	− 2.22351**	(0.45271)	− 3.21525**	(0.71094)
Divorced (yes/no)	− 1.07269**	(0.18138)	− 1.50778**	(0.28811)
Health shock	− 0.24891	(1.55831)	0.88095	(2.10432)
Health shock $\times$ Age	− 0.00361	(0.02858)	− 0.03768	(0.03912)
Health shock (m) $\times$ IAR (f)	0.10699^†^	(0.06348)	0.16736^†^	(0.09511)
Female partner				
Age	0.09752^†^	(0.05777)	0.20559*	(0.09610)
Age2	− 0.00083	(0.00062)	− 0.00187^†^	(0.00101)
Recently unemployed ($\ge$ 4months)	0.41808^†^	(0.23410)	0.42588	(0.30266)
Education (years)	− 0.05410^†^	(0.02852)	− 0.08063*	(0.03946)
Total unempl. exp. (months)	0.01627	(0.03455)	0.00974	(0.04809)
Child born prev. year	0.55716	(0.85101)	0.78713	(1.15319)
Non-German	0.65886*	(0.26487)	1.06867*	(0.43848)
IAR (f, 1000 EUR)	0.12097**	(0.02501)	0.19158**	(0.04088)
Civil servant (yes/no)	− 0.79377^†^	(0.44010)	− 0.79925	(0.64590)
Widowed before (yes/no)	− 2.18115**	(0.39938)	− 2.79371**	(0.56437)
Divorced (yes/no)	− 0.70003**	(0.16351)	− 1.06154**	(0.25927)
Health shock	1.23789	(1.45246)	2.30940	(1.91739)
Health shock $\times$ Age	− 0.03277	(0.03118)	− 0.05735	(0.04177)
Health shock (f) $\times$ IAR (m)	0.14589	(0.09483)	0.15190	(0.14745)
Couple-level variables				
Distance city center (km)	0.00216	(0.00300)	0.00419	(0.00451)
Property owners (yes/no)	− 0.09005	(0.15003)	− 0.23847	(0.21418)
No. of children	0.12243	(0.09369)	0.14881	(0.14281)
Main earner female (yes/no)	− 0.11363	(0.18716)	− 0.17097	(0.24705)
Couple’s income (1000 EUR)	− 0.01946**	(0.00514)	− 0.03031**	(0.00723)
Couple’s income (1000 EUR) squared	0.00002*	(0.00001)	0.00003**	(0.00001)
Constant	5.49094	(21.84722)	− 9.56203	(33.35433)
Survey year	− 0.00554	(0.01090)	− 0.00055	(0.01652)
logtime (pgmhaz) / time (xtlogit)	− 0.74968**	(0.09039)	− 0.14425**	(0.03293)
Gamma var. (pgmhaz) / lnsig2u (xtlogit)	0.66695**	(0.24458)	1.04373**	(0.37508)
Log (pseudo)likelihood	− 809.18104		− 836.34242	
*N*	2767		2767	

Hazard model specified in Eq. (1) and the logistic model in Eq. (2). The significance level symbols are ^†^ for 10%, * for 5%, and ** for 1%

Note that estimated coefficients are very similar for the two models showing no changes of sign and no major changes of significance across model specifications. Hence, the fact that the logistic model ignores the course of time may be of limited importance due to the rather short average spell length of approx. 5.4 years (although we find a significantly negative duration dependence for the Weibull specification of the hazard model, cf. the value of − 0.74968 for logtime which is significant at the 1 percent level). All in all, it turns out that mainly financial and health-related variables as well as family and professional status (rather than the socioeconomic variables included in the Becker model such as education, age, children, see “Marriage and health”) have a significant impact on (the timing of) marriages. For example, the IAR coefficient turns out to be a significantly positive predictor for (time to) marriage for both partners. Here, the parameter estimates reflect the increase in the expected logarithm (log) of the relative hazard in the order of 0.07 units (male) and 0.12 units (female) associated with a 1000 EUR increase in the respective IAR (in the event that there is no health shock and holding all other variables constant). For illustration, note that we arrive at hazard ratios after exponentiation of the above estimates, i.e., there is a 7% [$\exp (0.07)= 1.07$] increase in the expected hazard for each 1000 EUR increase in a male partner’s IAR (and, similarly, 13% for females).^16^

However, considering the IAR coefficient is not sufficient to assess our adverse selection hypothesis discussed in the section “Introduction”.^17^ Instead, we focus on the *health shock(m/f)* $\times$ *IAR(f/m)* crosswise interaction (where by crosswise we mean male health shocks with female IAR and vice versa to reflect the potential provision gap). This interaction is the basis for our empirical identification strategy. Economically, it reflects the supposed causal effect that IAR has on marriage hazards after people are exposed to health shocks and therefore consider the case of soon widowhood as more likely. For a discussion of potential endogeneity problems, see “Discussion”.

As expected, this interaction is significantly positive (at the 10% level) for male partner’s health shocks with female IAR which is also in line with their (still) prevailing role as breadwinner in different sex partnerships.^18^ Economically, this reflects that the reaction to male health shocks can be expected to differ for couples depending on their respective (female) IAR. In other words, a severe deterioration of male health is associated with a significant increase in marriage hazards for those couples in which female partners are at risk of losing a substantial part of their old-age provision if the male partner dies before marriage. The parameter estimates represent a significant 0.11 unit increase in the expected log marriage hazard (and a 12 percent increase in the expected hazard, where $\exp (0.11)= 1.12$) associated with each 1000 EUR of female IAR after a male partner’s health shock. Note that the corresponding interaction term for female partners is of the same order of magnitude, but insignificant which indicates a gender difference in the mechanism of action outlined above.

Other significant predictors of time to marriage include being a civil servant (in a permanent position) which roughly doubles the expected marriage hazard for male partners [$\exp (0.81)= 2.25$], but reduces the marriage hazard for female partners by approx. 55 percent [$\exp (-0.79)=0.45$]. Again, this result can be interpreted as an indication of the man’s still dominant provider role in marriages, which is probably linked to the income security of civil servant status. Interestingly, the influence reverses for women who work as civil servants, possibly reflecting a link between economic independence and lower propensity to marry in the sample. Similar arguments may explain the significantly positive coefficient for recent female unemployment which increases marriage hazards by approx. 50% [exp(0.42) = 1.52]. The same holds for the negative impact of female education reducing marriage hazards by approx. 5% per year [exp(− 0.05) = 0.95]. Both findings are in line with previous results from the literature, cf. “Main variables”.

Next, note that the fact that previous survivors’ pensions are lost in the event of remarriage is underlined by the significantly negative coefficient *married before* (and possibly related moral reasons against remarriage) showing an almost 90% reduction in marriage hazards for both male and female partners [$\exp (-2.22)= 0.11$ and $\exp (-2.18)= 0.11$]. Similarly, being divorced reduces marriage hazards significantly (by approx. 65% for males and 50% for females) where similar reasons may play a role (e.g., loss of existing maintenance claims against the former partner). Moreover, negative experiences with marriage per se may have a persistent effect and turn into a general reluctance to remarry. Marriage hazards are found to be significantly higher for non-Germans (both male and female) which can be discussed in terms of culturally different preferences for marriage and considerations of future maintenance, but this is not explored further in this study.

At the couple level, we find a significant u-shaped influence of income on marriage hazard. Note, however, that the negative linear coefficient alone will adequately capture the relationship for most couples as the minimum of the corresponding quadratic equation may be calculated to be EUR 487,000, an order of magnitude far outside the mean EUR 52,000 couple income in our sample.

As to the bottom part of Table 3, the potential influence of starting times in connection with the flow sampling approach and, hence, general time trends in marriage behavior are accounted for by incorporating survey year variables that turn out insignificant. The coefficient for *Gamma var.* (i.e., the variance of the gamma mixture distribution) is significant and, hence, provides evidence for unobserved heterogeneity in these data. Finally, the likelihood ratio statistic for the test of the model with vs. without gamma frailty also turns out to be significant (with a $\bar{\chi }^2_1$ statistic of 22.0623 at the 1% level of significance).

Next, we turn back to the logistic model to gain some additional insight into the interpretation of coefficient estimates. In particular, so-called marginal effects on the marriage probability for sample-average people may be calculated. To this end, Stata’s margins command is used to find that for average couples (in the sense of holding covariates constant at sample means) after a male health shock the marriage probability increases significantly by approx. 36% (with a s.e. of 0.105) for each additional thousand euros of the female partner’s IAR. Conversely, the corresponding marginal effect for female health shocks is approx. 27 percent (with a s.e. of 0.152).

### Discussion

The continuing rise of poverty among elderly singles poses a challenge to many Western countries and Western society. While policymakers rely on traditional solutions such as survivors’ pensions, inheritance tax benefits for the widowed or general social assistance these measures are far from sufficient to prevent old age poverty. In this context, this study asks whether mature cohabiting couples are willing to change their relationship status in response to health shocks due to economic considerations (windfall effects) for the likely survivor.

One of our core results is the significant (10% level) interplay of male health shocks with female IAR. It shows that the above economic considerations actually play a role in a couple’s marriage decision in this situation. For example, we find that health shocks for male partners increase marriage hazards by 36% (at average sample values) for a 1000 EUR increase in the female partner’s IAR, i.e., survivors’ pensions that would be lost in the unmarried state. From a (health) economic perspective, this result reflects the continuation of people’s rational behavior even in the face of (a serious) illness, cf. [46] for a discussion. In other words, health shocks may induce people to reevaluate their residual life expectancy, to assess their partner’s future economic situation and, hence, to estimate (at least roughly, to the best of their knowledge) the present value of their future income or pension under both scenarios, i.e., unmarried and married. Then, people tend to marry if it proves worthwhile under the new scenario. Economically, marriage in this situation can be interpreted as a kind of cost-free (pension or health) insurance, which people enter into only when they can be relatively sure (because of the health shock) that it will pay off.

Our results so far may be limited by the potential bias due to the transfer (inheritance) of wealth and real estate, since the inheritance tax also affects the net valuation differently for married and unmarried couples. We therefore included a variable AAR (cf. “Introduction”) in the robustness checks in Appendix 7, which captures the tax loss on unmarried vs. married inheritance of existing assets according to the law in force in the respective survey year.^19^ However, our results from Table 3 remain largely unchanged (even when interacting WAR with the health shock) and show that a marriage decision is influenced more by the income side (survivor’s pension) than by possible inheritance tax surcharges for unmarried people, see Appendix 7 for details. Possible reasons for this are manifold. First, unlike the survivor’s pension, there are many hypothetical assumptions to be made about inheritance and wealth^20^. Second, the coverage of assets in the SOEP is limited to single years, namely 2002, 2007, and 2012, which requires imputation and/or greatly reduces the size of our sample. Details are again given in Appendix 7. In addition, the survivor’s pension may play a larger role in the marriage decision than existing assets, simply by virtue of its provision character (social security). Particularly since the inheritance of assets (in addition to the compulsory portions to be taken into account) can also be arranged freely to a large extent among unmarried persons, so that *only* a possibly higher tax is to be paid.

A further caveat that should be discussed is the possible influence of the endogeneity problem on our results, which is well known in the literature on marital status and health, cf. [10, 30, 33]. In this context, omitted (unobservable) variables could lead to biased estimates of the impact of health on marital status. Overall, we consider the impact of this type of bias on our results to be small for the following reasons. First, following [10, 44], it can be argued that our results hold for rather severe health shocks, whereby these can also be considered increasingly exogenous with increasing severity (at most 5% of couple-years suffer a health shock, cf. “Main Variables”, which is further reduced to below 2% in the robustness checks in Appendix 7). Second, we consider a different problem from the bulk of the literature in which the endogeneity problem arises. Specifically, the selection of our sample (cf. “The data”) ensures that only current, unmarried relationships (from the date of moving in together) are considered, which reduces the probability of the occurrence of severe health-threatening relationship crises associated with subsequent marital status. Furthermore, our sample is not selective with regard to previous relationship crises (with previous partners). These are even explicitly captured by the variable *divorced* (i.e., a divorce before the current relationship). And third, if time-varying unobservable effects (e.g., in terms of poor relationship quality) in the current relationship might still have an impact on health shocks, then the potential bias associated with this would tend to underestimate our main effects (i.e., health shock $\times$ IAR).^21^. Thus, the interpretation of our results would remain unaffected (although, of course, it cannot be resolved whether the underestimation associated with the potential omitted variable bias would not still result in a significant effect for the female health shock $\times$ IAR (m) interaction). Nevertheless, fourth, we consider the influence of these unobservable relationship aspects on health and marriage to be small, because the panel approach already controls for time-constant unobservable effects, and the high average age will tend to involve rather relationship-experienced couples whose divorce history is also taken into account. And finally, in the robustness checks in Appendix 7, an objective and relatively rigorous (i.e., possibly only indirectly influenceable by relationship quality) health indicator is chosen in the form of hospital nights, which overall supports our results.

The abuse of marriage as an insurance against poverty in old age is not without risks, however, since extensive care obligations are associated with marriage, cf. “Main Variables”. The example of the UK, Denmark, or Sweden shows, however, that there are other possible policy solutions. Here, previous cohabitation may already entitle to a survivor’s pension. In this way, the survivor’s pension is linked more closely to the actual economic integration of the partners rather than legal status. At the same time, the couple is freed from the moral dilemma of marriage for maintenance, because the condition of cohabitation (if it is designed to be verifiable) allows less manipulation than formal marriage itself. Empirically, the insignificance of the interaction between health shocks and IAR for the low-income group demonstrated in Appendix 7 indicates that the strategy of picking windfalls in survivors’ pensions may be less a question of economic hardship (the lower income group, viz.), but rather a question of financial literacy (the higher income group).

Of course, several potential weaknesses in reliably identifying this type of adverse selection arise. For example, the data only allow an approximation for the measurement of health shocks (which are self-reported on a 1–5 scale). Nor are there any objective threshold values for the intensity of a health shock needed to be classified as potentially life-threatening. For this reason, extensive robustness checks were carried out in Appendix 7, showing that the results are generally robust to different definitions of health shocks. A similar reasoning applies to the measurement of IAR data, which include a hypothetical projection of uncertain income data into the future. In this regard, too, the stability of the results could be underlined by appropriate robustness checks.

Our results raise some interesting socioeconomic questions such as the different responses to health shocks of the male or female partner. The fact that the estimated interaction is significant only for male partner health shocks indicates that there is still a traditional male supply motive of marriage (since many men still seem to have the breadwinner’s role in the partnership). This result indicates that couples do not act as pure income maximizers in relation to the survivor’s pension, but depending on the context (of the health shock). Otherwise, the result should be independent of whether the health shock hits the male or female partner. A similar, male-dominated maintenance motive for marriage is also reflected in the result for civil servant status, its coefficient being positively significant for men (and thus associated with a higher probability of marriage). The corresponding coefficient is even negatively significant for women and may indicate their then greater economic independence from marriage.

Finally, there is the question of potential policy options in response to the results of this analysis. Here, the starting point is the unethical misuse of marriage for mere financial reasons from the perspective of the traditional welfare state, be it for financial security or to maximize income. For this reason, some unmarried couples may even feel criminalized if they marry (for whatever reason) shortly before the death of a partner. This is because the current rule considers the misuse of the marriage before the end of the waiting period as a standard (with sanctions such as the loss of the survivor’s pension) that must be disproved.

From a socioeconomic point of view, marriages for mature couples can be viewed as a pass-through of financial risks from the private to the public sector (where the latter is clearly a more suitable addressee for the pooling of such risks). Whether this form of quasi costless pension insurance should indeed be reserved to the married (by contrast with solid unmarried partnerships) is a question of future social and family policy settings and, of course, its difficult technical implementation to avoid overly excessive windfalls (e.g., spontaneously declared pseudo-partnerships to cream-skim financial benefits irrespective of any proven financial dependencies between two people).

The international response to these challenges varies widely. As mentioned above, some countries have extended the group of entitled persons to cohabiting partners. In France, however, waiting periods of up to 4 years of marriage may have to be fulfilled, whereas the waiting period in the U.S. is only 9 months. Even stricter rules apply in Switzerland, where only women (with few exceptions for men) receive a survivor’s pension after at least 5 years of marriage.

In the case of Germany, a rather uncontroversial policy measure to alleviate the deadweight problem could be a linear increase in the level of survivors’ pensions over the years of marriage. This is in contrast to the current all-or-nothing scheme after a 1 year waiting period. This proposal leaves open the question of a possible survivor’s pension entitlement also for stable (cohabiting) couples. However, a similar concept could apply here, which takes into account the duration and stability of cohabitation on a linear basis (and possibly includes a deduction compared to marriage to reflect the inherently greater risk of misuse of the concept). At the same time, recent debates about other alternative concepts to reduce old age poverty (such as a general basic income or minimum pensions) may help to mitigate the problem in the future—although the group of potential beneficiaries is expected to increase along with an aging generation of single households.

## Conclusion

This study investigated whether the likelihood of marriage for mature couples after a health shock depends on the expected level of survivors’ benefits. The econometric analysis based on a panel data set of 795 unmarried, mature couples in the years 1994–2017 shows a significant positive impact of the woman’s expected survivor’s pension after a severe health shock to the male partner. A quantitative interpretation of the results indicates that the probability of marriage is approx. 36% higher for every EUR 1000 more in the woman’s expected survivor’s pension.

From an economic point of view, the risk of survivors’ benefits is thus collectivized under asymmetrical information, since the health shock as the trigger for marriage cannot be reliably questioned after a waiting period of 1 year has been fulfilled. The said behavior can therefore be read as adverse selection. The statement of classic economic marriage models such as the Becker model, in which illness is modeled as a negative influencing factor or negative risk with regard to marriage, is thus turned into a positive economic incentive for a special situation in later life. To our knowledge, this is the first quantitative, empirical analysis on this topic.

The results also show clear gender differences, because the mechanism described above does not apply after the health shocks of women. This may point to traditional notions of the husband as the main provider in the marriage, but in any case, it represents a separate issue for future research. Other interesting research approaches include comparing international data, taking into account the different rules for survivors’ benefits.

In political terms, our results finally lead to the question of how the misuse of survivors’ pensions can be precisely defined and whether formal marriage can still be regarded as a contemporary prerequisite for receiving a survivor’s pension. For, as our analysis shows, the current regulation does not prevent any misplaced incentives, nor does it take into account the actual economic ties between the partners in an appropriate manner.

## Appendix: Robustness checks

To assess the robustness of our results, this section reviews some potentially critical assumptions of the baseline model estimated in the section “Main results”. To keep the results of the corresponding model variations as compact as possible, for each model variation denoted in column 1 of Table 4, only the two most relevant coefficients of interest for our research question are shown, namely the gender-specific interaction coefficients between health shocks and the partner’s IAR. The corresponding estimates are then presented in columns 2–5. According to column 1, only one model variation was implemented at a time. The respective model variations are explained in more detail below. Note, however, that statistical inferences resulting from such quasi-multiple post hoc tests should be interpreted with caution as a multiplicity correction is difficult to implement in empirical economic analyses, see, e.g.,  [5] for a discussion. The results are therefore to be considered as explorative and primarily indicative for future research.

**Table 4:** Robustness checks

	Male		Female	
	HS (m) $\times$ IAR (f)	(Std. Err.)	HS (f) $\times$ IAR (m)	(Std. Err.)
Age				
One partner older than 40	0.07407	(0.06340)	0.06671	(0.09296)
One partner older than 50	0.17862*	(0.07708)	0.11426	(0.09886)
One partner older than 55	0.26534*	(0.10653)	0.01135	(0.15852)
Health shock				
$\Delta \text{ CH}_t \le - 2$	0.17199*	(0.08490)	0.22683	(0.16617)
$\Delta \text{ CH}_t \le -1$	0.01052	(0.02846)	0.02020	(0.05947)
$\Delta \text{ SH}_t \le -5$	0.38053*	(0.15225)	− 0.02552	(0.34016)
$\Delta \text{ SH}_t \le -4$	0.32900**	(0.09787)	− 0.04813	(0.21936)
$\Delta \text{ HN}_t \text{ or } \Delta \text{ HN}_{t-1} \ge 3$	0.07542*	(0.03776)	0.05912	(0.06132)
$\Delta \text{ HN}_t \text{ or } \Delta \text{ HN}_{t-1} \ge 8$	0.14978*	(0.06668)	0.10957	(0.08866)
Income				
IAR (40% pension)	0.13204^†^	(0.07216)	0.14819	(0.10575)
IAR (60% pension)	0.10552*	(0.05056)	0.06195	(0.08298)
IARPV	0.02501^†^	(0.01319)	0.01901	(0.01978)
AAR	0.24033*	(0.11090)	0.04370	(0.14482)
High income	0.14879^†^	(0.08888)	0.12685	(0.11538)
Low income	0.09599	(0.21073)	− 0.10785	(0.20947)
Sample period				
$\le 2015$	0.21173**	(0.07678)	0.15738	(0.10852)
$\ge 1996$	0.11997^†^	(0.06218)	0.10323	(0.09471)
Model				
Normal frailty	0.12952*	(0.06282)	0.10378	(0.09695)
Cubic baseline hazard	0.13040*	(0.06641)	0.09810	(0.10016)
Nonparam. basel. haz.$^a$	0.12250*	(0.06158)	0.10004	(0.09213)

The corresponding model variation is given in the left column. Coefficient estimates for health shock $\times$ IAR interactions are given in the right columns (with significance level symbols as before). The superscript *a* indicates a model with normal frailty for reasons of numerical instability of the gamma model

To anticipate the results briefly, Table 4 generally shows broad agreement with the results from the section “Main results” in terms of magnitude and significance level, supporting the robustness of our basic model in various dimensions.^22^ In the first three rows of Table 4, the minimum age requirement of 45 years for at least one partner was challenged. It was shifted up and down in 5-year steps. As expected, the significance of the (male) coefficient for a sample that is rather young (40+) disappears, possibly due to a lack of relevance of the issue of provision for surviving partners, which is not perceived strongly enough at a rather young age. For the two older samples (50+ and 55+), however, the results are stable.

In the next part of the table, we assess the stability of our results with respect to the precise definition of the health shock. We make use of three alternative health state indicators provided in the SOEP, namely, as before, self-reported *current health* (CH, on a 1–5 scale) and self-reported *satisfaction with health* (SH, on a 0–10 scale). To provide a more objective measure of health status, we refer to *hospital nights* (HN, *How many nights altogether did you spend in the hospital last year?*). The first model variation, labeled $\Delta \text{ CH}_t \le -2$, represents a health shock if the self-reported current health status from the SOEP deteriorates by at least two units compared to the previous year. This, unlike the original definition of a health shock in the section “Main variables” (a two-point decrease in the previous year or the year before), does not include the penultimate year and thus turns out somewhat more stringent. Specifically, only 2.4% of men and 2.9% of women (with respect to all couple-year observations) now experience such a health shock. If the health shock requirement is softened somewhat further, so that already a worsening of self-reported current health by one unit defines a health shock ($\Delta \text{ CH}_t \le - 1$), then this is true for 18.0% of men and 18.4% of women, respectively, of all couple-year observations, which appears to be an overly soft definition. As expected, the estimated interaction terms in Table 4 are no longer significant. Next, we consider a definition of health shocks proposed by [44], namely a 5-point drop in *satisfaction with health* recorded in the SOEP on a 0–10 scale (labeled $\Delta \text{ SH}_t \le - 5$). With 1.4% of men and 1.8% of women, this is a rather strict definition, which, however, does not affect the stability of our results, but even increases the value of the estimated coefficient for men (at a significance level of 5%). This does not change even with a somewhat less stringent interpretation (drop of 4 points, $\Delta \text{ SH}_t \le - 4$), which now with 2.8% (men) and 3.5% (women) of couple-years is more in line again with the sample proportion from the original definition of a health shock in the section “Main variables”. To verify that the results are stable not only for self-reported but also objective health indicators, the number of hospital nights in the previous year was included as an indicator already used in the literature. The range proposed in the literature to define a health shock ranges from 3 nights, see [16], which seems somewhat low due to the rather mature sample, to 8 nights, see [37]. As in the section “Main variables”, we again include the last or penultimate year in the definition to adequately capture the possible waiting period in a marriage decision after a severe health shock. The health shock thus formed (labeled $\Delta \text{ HN}_t \text{ or } \Delta \text{ HN}_{t-1} \ge 3$, or 8) corresponds to approximately 14% (3 nights) to approximately 4% (8 nights) of the couple-years in the sample. Results remain stable over this range and comparable to those for self-reported health.

Next, the IAR definition which plays a central role in our empirical identification strategy has been modified to gauge its robustness with respect to (a) necessary simplifying assumptions in the calculation of the (expected) pension amount, (b) the consideration of a present value of the expected pension (weighted with the remaining life expectancy), and (c) the consideration of inheritable assets that have a different net value for married and unmarried couples due to the unequal inheritance tax treatment. With regard to (a), the future pension level in particular can be regarded as a critical assumption. The standard of a general pension level of 48% applied in the calculations of IAR in the sections “Social security for widow(er)s” and “Results and discussion” was therefore generously varied up and down within a corridor of 40–60%. The results proved to be robust. A further possible limitation of the strategy to calculate IAR is the consideration of annual values rather than (expected) lifetime-weighted present values for the IAR.^23^ Here, annual values may be subjectively weighted more for individuals with higher remaining life expectancy than for individuals with lower remaining life expectancy. To examine the possible influence of this effect on the results, the IAR values from section “Results and discussion” were converted to present values corresponding to residual life expectancy (IARPV). Residual life expectancy was determined as the difference between the respective ages to year-specific life expectancy at completed age. (The present value factor was set to 1, since future pension increases were also ignored in our analysis. However, a sensitivity analysis shows that the results remain stable even for broad variations in this value.) Using this approach, the significance level of the interaction of the male health shock with IARPV (f) remains significant at the 10% level, while the interaction for a female health shock with IARPV (m) remains insignificant as before, cf. Table 4. To test the effect of assets (such as real estate and financial investments) on the propensity to marry after health shocks, cf. the section “Discussion”, an AAR variable was formed that, analogous to the IAR, captures the loss of wealth in an inheritance among unmarried vs. married partners. The background to this is the favorable tax treatment of married couples under the inheritance tax (higher allowances, lower tax rates). Some assumptions have to be made, such as the amount of the intended inheritance, which is unknown in the individual case. Since this is a robustness check of our IAR interaction, we assumed here the extreme case that the entire assets recorded in the SOEP are to be transferred to the partner. In calculating the AAR, we took into account the allowances and average tax rates in each case before and after the 2009 inheritance tax reform, as well as the tax-free inheritability of owner-occupied real estate among spouses. Since assets were not recorded annually in the SOEP, but only in 2002, 2007, and 2012, imputation was required for the missing years, which was implemented here by the last observation carried forward or backward (depending on which point in time is closer) method. The results show that this does not change the significance of the HS $\times$ IAR interactions (shown in the AAR row of Table 4). The HS $\times$ AAR interactions themselves are not reported in Table 4. These are insignificant and are − 0.02356 (0.02997) for men and − 0.00031 (0.00345) for women. In addition, we will address the question of whether the results can be considered stable within different income groups. To this end, the sample is split into a higher and lower income group (using the sample median of approx. EUR 42,000 at the couple level). The estimated interaction coefficient HS (m) $\times$ IAR (f) remains significant only for the high income group, cf. the section “Discussion”.

Finally, we included some more technical modifications all of which also underline the robustness of the base model’s results. A shortening of 2 years each at the beginning and end of the sample period was considered to exclude possible boundary effects in the choice of the sample. The assumption of a parametric baseline hazard (Weibull, cf. Sect. “Econometric model”) was relaxed in favor of a cubic and fully nonparametric specification, respectively, see [25] for details. The results also proved to be robust when the gamma frailty term was replaced by a normal distribution. Note that all of the above changes were also tested in the logit model with similar results.

## Footnotes

1 Note that the SOEP sample used in this study shows a comparable poverty rate for 2008 of 19% for 40–85-year-old unmarried women and 14% for widows of the same age group, according to the poverty threshold of 60% of the median of the equivalized income of the total population used in [15]. For unmarried men in the same age groups, the figures are 16% and 8% for widowers.

2 German law requires 1 year.

3 In the data, we will approximate this criterion by focusing on unmarried couples who live in the same household.

4 Further results related to this question are reported by [29] for U.S. data, where mood, anxiety, or addictive disorders are reported as significant (with the exception of social and simple phobia) predictors in survival models of marriage duration to divorce.

5 Note that [7] defines *marriage* as sharing the same household.

6 A major amendment of the law became effective in 2002, where in particular the level of survivor’s benefits was reduced to 55% (previously 60%) of the deceased spouse’s actual or calculated pension.

7 The legislator assumes that such marriages for maintenance intentionally skim off the pension income of the spouse and thus contradict the actual goals of the marriage considered worthy of protection (§46 SGB VI).

8 The latter condition stands in contrast to a less stringent legislation, e.g., in Canada or in the U.S. where payments may be only temporarily interrupted according to the recipient’s age, see, e.g.,  [9].

9 Note that inclusion in the survey is not bound to German citizenship.

10 This is achieved by dropping all individuals with partners living in the same household in the first wave of their SOEP participation. Thereby the sample is restricted to each person’s first stable partnership spell (and first marriages, if applicable) after entering the SOEP. Furthermore, only complete sequences of observations are considered, i.e., for temporal dropouts with individuals returning to the SOEP at a later date we only make use of the spell until her or his first exit.

11 Note that we exclude same-sex partnerships, as formal same-sex marriage was not established in Germany until 2017 (with registered partnerships introduced in 2001).

12 This number is later also found in the last row of Table 3, since the $N=2767$ observations in the model correspond to the number of couple-years.

13 Whereas this tends to overestimate objective life expectancy for reasons of hedonic adaptation after health shocks, cf. [6, 43].

14 Some more flexible specifications will be discussed in Appendix 7.

15 As an alternative, we consider a normal distribution for $u_i$ in Appendix 7.

16 Recall that the coefficient for female IAR reflects the male partner’s income contribution that is lost in the event of his death before marriage and vice versa.

17 For example, IAR’s significantly positive coefficient may be driven by reasons regardless of the respective partner’s state of health (e.g., by different motives to bind a partner with a high current income by marriage) and, hence, regardless of any deadweight effects that play with the thought of future survivor’s pension payments.

18 This is a result that is often described as *marginally* significant at the 10% level. However, alternative model specifications in Appendix A show significant coefficients at the 5% level, although we carefully point out the multiple test problem for their interpretation.

19 Statistically, this omitted variable may bias the IAR coefficients, although the sample revealed only very low correlations (smaller than 0.05 in magnitude) between IAR and AAR.

20 To give one example: A tax on the inheritance of owner-occupied residential property is only due if the property is occupied by the heir for at least 10 years after the inheritance

21 This is shown by a brief plausibility check of the correlations: Health shocks would certainly be positively correlated with poor relationship quality (i.e., the omitted variable), and health shocks themselves would in turn be negatively correlated with the outcome marriage, which as a result tends to underestimate the effect of health shocks on marriage

## References

[1] Aassve A, Burgess S, Chesher A, Propper C (2002). Transitions from home to marriage of young Americans. J. Appl. Economet..

[2] Ahn, N., Mira, P.: Job bust, baby bust?: Evidence from Spain. In: Zimmermann K., Vogler M. (eds.) Family, household and work. Population economics, pp. 389–405. Springer, Berlin (2003). 10.1007/978-3-642-55573-_19

[3] Alm J, Whittington LA (1995). Income taxes and the marriage decision. Appl. Econ..

[4] Alm J, Whittington LA (1997). Income taxes and the timing of marital decisions. J. Public Econ..

[5] Athey S, Imbens GW (2017). The state of applied econometrics: causality and policy evaluation. J. Econ. Perspect..

[6] Baji P, Bíró A (2018). Adaptation or recovery after health shocks? Evidence using subjective and objective health measures. Health Econ..

[7] Becker GS (1973). A theory of marriage: part I. J. Political Econ..

[8] Becker GS (1974). A theory of marriage: part II. J. Political Econ..

[9] Brien MJ, Dickert-Conlin S, Weaver DA (2004). Widows waiting to wed? (Re)Marriage and economic incentives in social security widow benefits. J. Hum. Resour..

[10] Bünnings, C., Hafner, L., Reif, S., Tauchmann, H.: In sickness and in health?. Empirical evidence from Germany, Health shocks and relationship breakdown (2020)

[11] De Angelis, R., Sant, M., Coleman, M.P., Francisci, S., Baili, P., Pierannunzio, D., Trama, A., Visser, O., Brenner, H., Ardanaz, E., Bielska-Lasota, M., Engholm, G., Nennecke, A., Siesling, S., Berrino, F., Capocaccia, R.: Cancer survival in Europe 1999–2007 by country and age: results of EUROCARE-5—a population-based study. Lancet Oncol. **15**(1), 23–34 (2014). 10.1016/S1470-2045(13)70546-1 10.1016/S1470-2045(13)70546-1 24314615

[12] Deutsche Rentenversicherung. Statistik der Deutschen Rentenversicherung—Rentenbestand am 31.12.2010 [Statistics of the Deutsche Rentenversicherung—Pension portfolio on 31.12.2010]. Technical report, Deutsche Rentenversicherung, Berlin, 2011

[13] Dickert-Conlin S (1999). Taxes and transfers: their effects on the decision to end a marriage. J. Public Econ..

[14] Diekmann A (1989). Diffusion and survival models for the process of entry into marriage. J. Math. Sociol..

[15] Engstler H, Wolf T, Motel-Klingebiel A (2011). Die Einkommenssituation und -entwicklung Verwitweter in Deutschland [The income situation and development of widowers in Germany]. Vierteljahrshefte zur Wirtschaftsforschung.

[16] Garcia-Gomez, P., van Kippersluis, H.O. O’Donnell, H., van Doorslaer, E.: Effects of health on own and spousal employment and income using acute hospital admissions (2011). http://nbn-resolving.de/urn:NBN:nl:ui:15-1765/26527

[17] Goebel, J., Grabka, M.M.: Zur Entwicklung der Altersarmut in Deutschland [On the development of old-age poverty in Germany]. DIW Wochenbericht **78**(25), 3–16 (2011). 10.3790/vjh.80.2.101.. http://ideas.repec.org/a/diw/diwwob/78-25-1.html

[18] Griffin B, Loh V, Hesketh B (2013). A mental model of factors associated with subjective life expectancy. Soc. Sci. Med..

[19] Gutiérrez-Domènech M (2008). The impact of the labour market on the timing of marriage and births in Spain. J. Population Econ..

[20] Hagan R, Jones AM, Rice N (2008). Health shocks and the hazard rate of early retirement in the ECHP. Swiss J. Econ. Stat..

[21] Harrington Meyer, M., Street, D., Quadagno, J.: The impact of family status on income security and health care in old age: a comparison of western nations. Int. J. Sociol. Soc. Policy **14**(1/2), 53–83 (1994). 10.1108/eb013186

[22] Hiekel, N.: The different meanings of cohabitation across Europe: How cohabiters view their unions and differ in their plans and behaviors. Amsterdam University Press, Amsterdam (2014)

[23] Holland, J., Perelli-Harris, B., Andersoon, G.: ’Does Marriage Matter?’ Revisited: the stability, fertility and mortality of the Swedish 1989 Marriage Boom cohort. 2017. http://hdl.handle.net/1765/116455

[24] Jenkins SP (1995). Easy estimation methods for discrete-time duration models. Oxford Bull. Econ. Stat..

[25] Jenkins SP (1998). Discrete time proportional hazards regression. Stata Tech. Bull..

[26] Jenkins, S.P.: Survival analysis. 2005

[27] Jones, A.M., Rice, N., Bago d’Uva, T., Balia, S.: Applied Health Economics. Routledge, London and New York (2013). 10.4324/9780203102411

[28] Joung IM, Van De Mheen HD, Stronks K, Van Poppel FW, Mackenbach JP (1998). A longitudinal study of health selection in marital transitions. Soc. Sci. Med..

[29] Kessler RC, Walters EE, Forthofer MS (1998). The social consequences of psychiatric disorders, III: probability of marital stability. Am. J. Psychiatry.

[30] Kohn JL, Averett SL (2014). The effect of relationship status on health with dynamic health and persistent relationships. J. Health Econ..

[31] Kooij D, Van De Voorde K (2011). How changes in subjective general health predict future time perspective, and development and generativity motives over the lifespan. J. Occupational Org. Psychol..

[32] Kortmann, K.: Alterssicherung im 21. Jahrhundert und deren Erforschung mit Mikrodaten—Der Beitrag der Untersuchungen zur Alterssicherung in Deutschland (ASID) [Old-age security in the 21st century and its study with microdata]. Deut. Rentenversicherung **6**5(2), 286–300 (2010)

[33] Lillard LA, Panis CWA (1996). Marital status and mortality: the role of health. Demography.

[34] Lillard LA, Brien MJ, Waite LJ (1995). Premarital cohabitation and subsequent marital dissolution: a matter of self-selection?. Demography.

[35] Lundberg S (2012). Personality and marital surplus. IZA J. Labor Econ..

[36] Meyer BD (1990). Unemployment insurance and unemployment spells. Econometrica.

[37] Mühlenweg AM, Westermaier FG, Morefield B (2016). Parental health and child behavior: evidence from parental health shocks. Rev. Econ. Household.

[38] Newman, J.L., McCulloch, C.E.: A hazard rate approach to the timing of births. Econometrica **52**(4), 939 (1984). 10.2307/1911192. https://www.jstor.org/stable/1911192?origin=crossref 12266380

[39] Ng BP, Jensen GA (2018). Health shocks and initiation of use of preventive services among older adults. J. Appl. Gerontol..

[40] Oppenheimer, V.K.: Women’s employment and the gain to marriage: the specialization and trading model. Ann. Rev. Sociol. **23**, 431–53 (1997) 10.1146/annurev.soc.23.1.431 12348280

[41] Oppenheimer, V.K.: Cohabiting and marriage during young men’s career-development process. Demography **40**(1), 127–149 (2003). 10.1353/dem.2003.0006 10.1353/dem.2003.0006 12647517

[42] Pevalin DJ, Ermisch J (2004). Cohabiting unions, repartnering and mental health. Psychol. Med..

[43] Pinquart M (2001). Correlates of subjective health in older adults: a meta-analysis. Psychol. Aging.

[44] Riphahn RT (1999). Income and employment effects of health shocks: a test case for the German welfare state. J. Population Econ..

[45] Robert Koch Institut. Zentrum für Krebsregisterdaten [Center for Cancer Registry Data], 2018 10.1007/s00103-011-1361-7 22015795

[46] Sally, D.: Confronting the Sirens: rational behavior in the face of changing preferences. J. Inst. Theor. Econ. **1**56(4), 684–714 (2000)

[47] Schmidt L (2008). Risk preferences and the timing of marriage and childbearing. Demography.

[48] SOEP. Socio-Economic Panel (SOEP), data for years 1984-2017, version 34. (2019)

[49] Spivey C (2010). Desperation or desire? The role of risk aversion in marriage. Econ. Inq..

[50] StataCorp. Stata Statistical Software: Release 13 (2013)

[51] Statistisches Bundesamt (Destatis). Verbraucherpreisindizes für Deutschland—Lange Reihen ab 1948 (Dezember 2018) [Consumer Price Indices for Germany—Long series from 1948 (December 2018)] (2019)

[52] Sundmacher, L.: The effect of health shocks on smoking and obesity. Eur. J. Health Econ. **13**(4), 451–60 (2012). 10.1007/s10198-011-0316-0. http://www.ncbi.nlm.nih.gov/pubmed/21559942 10.1007/s10198-011-0316-0 21559942

[53] Trussell J, Bloom DE (1983). Estimating the co-variates of age at marriage and first birth. Popul. Stud..

[54] VDEK. Finanzielle Belastung eines Pflegebedürftigen in der stationären Pflege [Financial burden of a person in need of inpatient care] (2019). https://www.vdek.com/presse/daten/f_pflegeversicherung.html

[55] Wooldridge J (2010). Econometric analysis of cross section and panel data.

